# Common Cancers in Karachi, Pakistan: 2010-2019 Cancer Data from the Dow Cancer Registry

**DOI:** 10.12669/pjms.36.7.3056

**Published:** 2020

**Authors:** Muhammad Asif Qureshi, Saeed Khan, Shaheen Sharafat, Mohammed Saeed Quraishy

**Affiliations:** 1 Prof. Dr. Muhammad Asif Qureshi, MBBS, PhD (Glasgow-UK), MA (IR), Postdoc (Germany), CHPE. Department of Pathology, Dow International Medical College, Dow University of Health Sciences Karachi, Karachi - Pakistan; 2 Prof. Dr. Saeed Khan, MSc, PhD, Postdoc (USA). Department of Pathology, Dow International Medical College, Dow University of Health Sciences Karachi, Karachi - Pakistan; 3 Prof. Dr. Shaheen Sharafat, MBBS, M.Phil., PhD. Department of Pathology, Dow International Medical College, Dow University of Health Sciences Karachi, Karachi - Pakistan; 4 Prof. Dr. Mohammed Saeed Quraishy, FCPS, FRCS. Department of Surgery & Vice Chancellor, Dow International Medical College, Dow University of Health Sciences Karachi, Karachi - Pakistan

**Keywords:** Cancer registry, Cancer patterns, Karachi, Pakistan

## Abstract

**Objectives:**

To present 2010-2019 cancer data from the Dow Cancer Registry representing all districts of Karachi (~17.4 million).

**Methods:**

The study was conducted at the Dow University of Health Sciences. After ethical approval, the Dow Cancer Registry was established at the largest government-run diagnostic and reference center of Karachi (Dow Labs). All cancers registered during 2010-2019 were analyzed. Patients >18years of age were labeled as adults while those with ages ≤18years were classified as children/young adults.

**Results:**

During 2010-2019, a total of 22,858 cancers were registered. Of these, 9,112(39.9%) cancers were diagnosed in males while 13,746(60.1%) in females. Incidence rates for all cancers (all ages) were 108/1,00,000 for males and 188.6/1,00,000 for females. In adult males, cancer of lip and oral cavity was the most frequently diagnosed cancer (33.6%), followed by non-melanoma –skin-cancer (NMSC) (7.2%), oesophagus (6.8%), colorectum (6.7%) and stomach (4.9%). In adult females, breast cancer was the most frequently recorded malignancy (53.2%), followed by cancers of lip and oral cavity (10.4%), oesophagus (5.3%), colorectum (3.3%) and NMSC (3%). In children, most common malignancy was that of brain and nervous system (15.3%), followed by Hodgkin’s lymphoma (14.2%), colorectum (8.1%), endocrine-&-related organs (8%) and non-Hodgkin’s lymphoma (7.8%)

**Conclusion:**

Cancers of lip and oral cavity and breast cancer were the most common malignancies in males and females respectively. In paediatric group, cancers of brain and nervous system were most common. Alarmingly, Karachi males have highest ASR of cancers of lip and oral cavity compared to any other city of Pakistan.

## INTRODUCTION

National-level cancer data have never been published from Pakistan.^1^ Cancer statistics outlined in the Globocan-2018 report for Pakistan are extracted from the Punjab Cancer Registry (PCR), which being a provincial registry, does not represent national level cancer numbers.^2^,^3^ Establishment of the National Cancer Registry (NCR) at the Pakistan Medical Council (now called the Pakistan Health Research Council) during 1970s was a commendable step by the Government of Pakistan.^4^,^5^ Another notable regional cancer registry was the Karachi Cancer Registry (KCR), that was established during mid-1990s and published several reports describing cancer burden for the region of Karachi South (1/6 district regions of Karachi).^6^ However, after the demise of KCR founder, Dr. Yasmin Bhurgri, the KCR largely became non-functional until recently when it was revived by contribution from some of the major stake holders (including the Dow Cancer Registry) of cancer diagnostics and registration in the city. The revived KCR has recently started data collection and is in the process of publishing its first report.^7^ Despite these commendable regional efforts, there are high chances that many of the cancer patients are not even registered in Pakistan due to absence of an effective national level cancer registration system. It is therefore extremely important to regularly generate good quality regional cancer data.

With this context, we established the Dow Cancer Registry at the largest public-sector diagnostic & reference laboratory of Karachi – the Dow Labs.^8^ Our cancer registry registers patients from all over the city via the Dow labs which has >25 collection points distributed throughout the city. Cancer data presented herein are therefore large-scale high quality regional data representative of all districts of Karachi (~17.4 million).

This report details cancer burden in Karachi during 2010-2019. These large-scale data will therefore significantly contribute/reflect towards identifying cancer burden in Karachi and will facilitate policy makers to devise appropriate strategies regarding cancer prevention and control in Karachi, Pakistan.

## METHODS

This epidemiological study was conducted at the Dow University of Health Sciences (DUHS), Karachi. After institutional ethical approval (approval#:IRB-459/DUHS/-14, renewal#:IRB-1667/DUHS/Approval/2020), the Dow Cancer Registry was established at the largest government-run diagnostic and reference laboratory, the Dow Labs of the DUHS, which registers cancer patients/samples from all over Karachi via its >25 collection points distributed throughout the city. First report from our registry described cancer patterns in Karachi during 2010-2015.^8^ We now present ten years (2010-2019) cancer data from our registry.

Data collection and indexation was undertaken as previously described.^8^ Briefly, all cancers registered at the Dow Cancer Registry were digitally archived in the in-house built software and were coded using the international classification of disease (ICD-10, version:2019).^9^ Subsequently, all data were indexed in MS Excel and SPSS for further analyses. Only those patients were registered for whom the cancer diagnosis was backed up by histopathological report. For analyses purposes, patients with >18years of age were considered as adults while patients ≤18 years were labeled as children/young adults.

In order to calculate age standardized rates (ASR) for cancers, age specific Person-years of males and female population at risk in Karachi were calculated using data projected from the 1998 census (as reported by the Sindh and Pakistan Bureau of Statistics) by incorporating an inter-census growth rate of 3.5% (Fig.1A).^10^,^11^ Frequencies of cancers were listed as number and percentages. Overall ASR was calculated for all cancers including as well as excluding NMSC. ASR world data were extracted from the Globocan 2018 report.^3^

## RESULTS

A total of 22,858 cancers were registered during the 10-year period from January 1, 2010 through to December 31, 2019. Of these, a total of 13,746 (60.1%) were diagnosed in females and 9,112 (39.9%) were diagnosed in males. Age wise, 97.4% cancers were diagnosed in adults while 2.6% cancers were diagnosed in children/young adults. Frequencies, percentages and ASR for all cancers in both genders and all ages are shown in Table-I. ASR of top five cancer sites in males and females are shown in Fig.1B and Fig.1C respectively. Overall (in both genders, all ages), breast cancer was the most common cancer (31.8% of all cancers) followed by cancers of lip and oral cavity (19.2%), oesophagus (5.8%), colorectum (7.0%) and non-melanoma skin cancers (NMSC) (6.9%).

**Table-I:** Frequency, percentages and age standardized incidence rates (ASR) of malignancies in males and females (all ages) in Karachi, 2010-2019 (N=22858).

Site		Both Gender			Male			Female			ASR World

	ICD-10-	n	%	ASR	n	%	ASR	n	%	ASR	
Breast	C50	7270	31.8	46.2	73	0.8	0.9	7197	52.4	98.7	46.3
Lip and oral cavity	C00-06	4400	19.2	28.0	2986	32.8	35.4	1414	10.3	19.4	4.0
Oesophagus	C15	1319	5.8	8.4	599	6.6	7.1	720	5.2	9.9	6.3
Colorectum	C18-20	1094	4.8	7.0	625	6.9	7.4	469	3.4	6.4	19.2
NMSC	C44	1078	4.7	6.9	656	7.2	7.8	422	3.1	5.8	10.1
Stomach	C16	652	2.9	4.1	433	4.8	5.1	219	1.6	3.0	11.1
Larynx	C32	538	2.4	3.4	400	4.4	4.7	138	1.0	1.9	2.0
Lung	C34	496	2.2	3.2	374	4.1	4.4	122	0.9	1.7	22.5
NHL	C82-85	487	2.1	3.1	294	3.2	3.5	193	1.4	2.6	5.7
Liver	C22	458	2.0	2.9	257	2.8	3.0	201	1.5	2.8	9.3
Corpus uteri	C54	384	1.7	2.4	0	0.0	-	384	2.8	5.3	8.4
Brain, nervous system	C70-72	362	1.6	2.3	222	2.4	2.6	140	1.0	1.9	3.5
Ovary	C56	338	1.5	2.1	0	0.0	-	338	2.5	4.6	6.6
Hodgkin’s lymphoma	C81	289	1.3	1.8	186	2.0	2.2	103	0.7	1.4	1.0
Urinary bladder	C67	253	1.1	1.6	194	2.1	2.3	59	0.4	0.8	5.7
Prostate	C61	242	1.1	1.5	242	2.7	2.9	0	0.0	-	29.3
Small intestine	C17	236	1.0	1.5	140	1.5	1.7	96	0.7	1.3	Ӿ
Thyroid	C73	215	0.9	1.4	53	0.6	0.6	162	1.2	2.2	6.7
Cervix uteri	C53	175	0.8	1.1	0	0.0	-	175	1.3	2.4	13.1
Salivary gland	C07-08	148	0.6	0.9	90	1.0	1.1	58	0.4	0.8	0.6
Nasopharynx	C11	146	0.6	0.9	95	1.0	1.1	51	0.4	0.7	1.5
Pharynx	C9-10	143	0.6	0.9	100	1.1	1.2	43	0.3	0.6	2.0
Bone	C40-41	139	0.6	0.9	81	0.9	1.0	58	0.4	0.8	Ӿ
Anal Canal	C21	134	0.6	0.9	84	0.9	1.0	50	0.4	0.7	0.5
Omentum, mesentry	C48	130	0.6	0.8	38	0.4	0.5	92	0.7	1.3	Ӿ
Gall bladder	C23-24	128	0.6	0.8	39	0.4	0.5	89	0.6	1.2	2.3
Testis	C62	81	0.4	0.5	81	0.9	1.0	0	0.0	-	1.7
Pancreas	C25	48	0.2	0.3	27	0.3	0.3	21	0.2	0.3	4.8
Melanoma of skin	C43	44	0.2	0.3	26	0.3	0.3	18	0.1	0.2	3.1
Others	-	1431	6.3	9.1	717	7.9	8.5	714	5.2	9.8	-
Total (all sites including NMSC)	-	22858		145.4	9112		108.0	13746		188.6	197.9
Total (all sites excluding NMSC)	-	21780		138.5	8456		100.3	13324		182.8	187.8

*not reported in the Globocan 2018

In adult males, the most frequently diagnosed cancer was the cancer of lip and oral cavity (33.6% of male cancers), followed by NMSC (7.2%), oesophagus (6.8%), colorectum (6.7%), and cancer of stomach (4.9%) (Table-II).In adult females, breast cancer was the most frequently recorded malignancy (53.2%), followed by cancers of lip and oral cavity (10.4%), oesophagus (5.3%), colorectum (3.3%) and NMSC (3.0%).

**Table-II:** Comparison of percentages amongst Dow Cancer Registry data (2010-2019) and other regional registries in Pakistan.

Site	ICD-10-	Dow Cancer Registry	Karachi Cancer Registry^*^		PAEC^*^	Punjab Cancer Registry

		2010-2019	1995-1997^6^	1998-2002^12^	2015-2017^15^	2014-2018^2^
ADULT MALES		n=8781	n=2160		n=14021	n=12208
Lip and oral cavity	C00-06	33.6	10.0	22.5	25.9	8.2
NMSC	C44	7.2	-	-	-	-
Oesophagus	C15	6.8	4.4	6.3	-	-
Colorectum	C18-20	6.7	3.9	7.8	5.6	7.2
Stomach	C16	4.9	2.8	6.0	-	-
Larynx	C32	4.5	5.8	11.8	4.6	-
Lung	C34	4.2	12.6	25.5	8.2	7.8
NHL	C82-85	3.0	4.6	-	3.7	7.4
Liver	C22	2.9	3.7	5.3	8.1	5.4
Prostate	C61	2.7	3.1	9.8	3.2	8.5
ADULT FEMALES		n=13487	n=2108		n=14732	n=16549
Breast	C50	53.2	33.1	69.1	32.2	45.0
Lip and oral cavity	C00-06	10.4	8.0	20.4	11.4	3.9
Oesophagus	C15	5.3	3.4	8.6	4.5	-
Colorectum	C18-20	3.3	3.1	5.2	3.1	3.6
NMSC	C44	3.0	-	-	-	-
Corpus uteri	C54	2.8	3.1	5.0	3.2	4.4
Ovary	C56	2.3	6.5	7.8	5.3	4.2
Stomach	C16	1.6	1.7	4.0	-	-
Liver	C22	1.5	2.0	4.0	-	2.6
NHL	C82-85	1.3	2.3	-	2.7	2.3
CHILDREN & YOUNG ADULTS		n=590				n=2126
Brain, nervous system	C70-72	15.3	-	-	-	12.6
Hodgkin’s lymphoma	C81	14.2	-	-	-	9.9
Colorectum	C18-20	8.1	-	-	-	-
Endocrine & related organs	C74-75	8.0	-	-	-	4.9
Non-Hodgkin’s lymphoma	C82-85	7.8	-	-	-	8.9
Orbit	C69	7.5	-	-	-	3.6
Bone	C40-41	5.6	-	-	-	4.9
Lip and oral cavity	C00-06	5.3	-	-	-	-
Breast	C50	4.4	-	-	-	-
Ovary	C56	3.7	-	-	-	3.9

* adult and paediatric population were not analyzed separately in these reports.

In children/young adults, the most frequently diagnosed cancer was cancers of brain and nervous system (15.3%), followed by Hodgkin’s lymphoma (14.2%), colorectal carcinoma (8.1%), cancers related to endocrine and related organs (8.0%) and Non Hodgkin’s lymphoma (7.8%) .


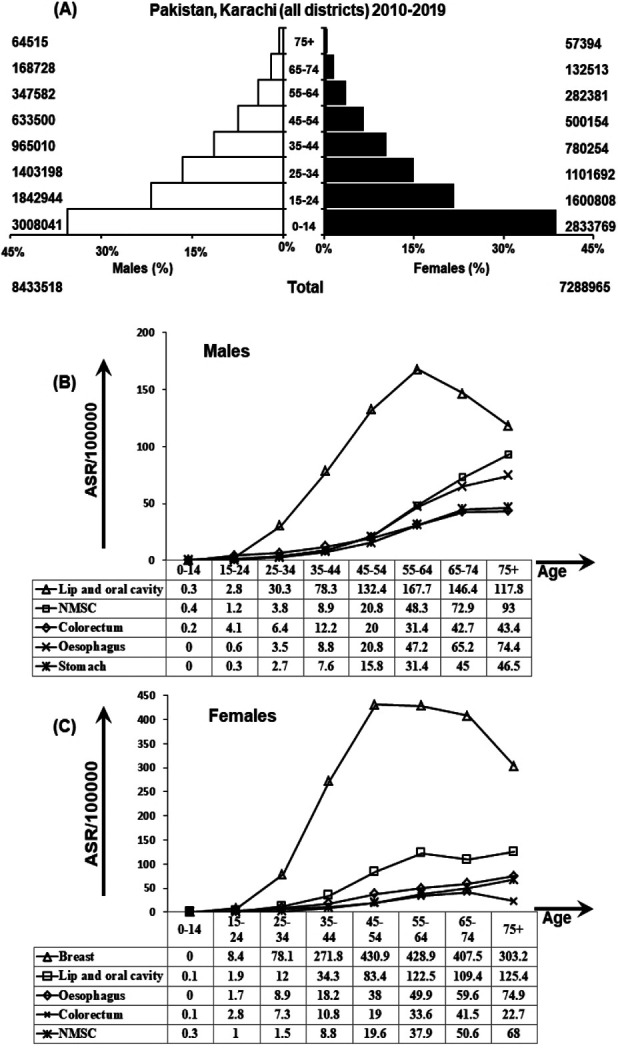

- Person-years of population at risk by gender and age.
  Projected population taken from census 1997 data were used to plot person-years of population at risk in both gender and all age groups.
- Age-standardized-incidence-rates for top five cancer sites in males.
  ASR was calculated using age specific population and age wise distribution is presented in top five malignancies in males.
- Age-standardized-incidence-rates for top five cancer sites in females.
  ASR was calculated using age specific population and age wise distribution is presented in top five malignancies in females.
  (NMSC: non melanoma skin cancer)

We also compared our data with cancer numbers from the (old) Karachi Cancer Registry, Pakistan-Energy-Commission (PAEC); 2015-2017, and the Punjab Cancer Registry during; 2014-2018 (Table-II). While our data, by-enlarge, show patterns similar to other regional reports from Karachi, there were considerable differences compared to data from Punjab, particularly the very high burden of cancers of lip and oral cavity in Karachi.

## DISCUSSION

Overall, we report breast cancer as the most common cancer in both genders with cancer of the lip and oral cavity as the most common cancer in males and second most common cancer in females. High prevalence of cancers of lip and oral cavity in Pakistan has been reported from other regional reports as well.^6^,^8^,^12^-^15^

Alarmingly, we report highest ASR of cancers of lip and oral cavity in males of Karachi compared to any other city of Pakistan. Notably, many of the patients with oral cancers have chronic history of tobacco consumption,^16^ which takes several different forms including (1) smoking in the form of “cigarettes”, “shisha” (tobacco smoking with a tube immersed in fragrant liquid, “bidi” (dried rolled tobacco leaves) (2) chewing in the form of “betel quids” (paan), “beetle-nuts”, “naswar” (moist powdered tobacoo kept in mouth for several hours) and “gutka” (mixture of various things including areca nuts, lime, paraffin wax, tobacco and catechu). Of these, consumption of gutka and naswar is very high in Karachi as compared to other cities of Pakistan.^17^ These are kept in mouth for several hours, thus exposing the mucosa to the chemicals and thus increased chances of developing oral submucosal fibrosis and oral cancers.^18^ Importantly, knowledge regarding the detrimental effects of smokeless tobacco is poor in Karachi.^19^ Therefore, relevant intervention by concerned authorities can play a vital role in controlling this preventable cancer.

Breast cancer was the most common malignancy in females and these findings are in line with other national and international reports.^3^,^13^-^15^ While breast cancers are by and large non-preventable, various socioeconomic factors such as increased use of contraceptives, lack of breastfeeding, and obesity are known risk factors.^20^ Moreover, genetic factors, such as BRCA1 and BRCA2 mutations, have been identified in Pakistani patients.^21^ Nevertheless, detailed epidemiological and mechanistic investigations are scarce to delineate risk factors for breast cancer pathogenesis in Pakistan.

Oesophageal cancer was amongst the top cancers in both genders. This is consistent with our previously published report.^8^ High burden of oesophageal cancer has been previously reported in Quetta city of Pakistan which is in close proximity to Iran – one of the cities in the well-defined “Asian-oesophageal-cancer-belt” (Iran, Iraq, Turkey, USSE, China and Mongolia).^22^ Oesophageal cancer was also very common in Afghan population coming to PAEC for their treatment.^15^ It needs to be investigated if there is an increasing trend of consuming dietary risk factors for oesophageal carcinoma including alcohol, hot beverages, smoked meet (BBQ) in Pakistan.

Colorectal cancer was amongst the most commonly detected cancers in both genders. This is consistent with our previous report and other regional reports.^8^,^13^-^15^ While this cancer has traditionally been prevalent in developed countries, its high prevalence in Pakistan is alarming and may have been caused by increasing adaptation to westernized diet and life style in Pakistan.^23^

We report non melanoma skin cancers (NMSCs) amongst the top cancers in both genders in Karachi. Since Karachi is located nearer to the equator (latitude 24.8^º^N and longitude 67.1^o^E) as compared to other parts of Pakistan, it is possible that its proximity to the equator imposes higher risk of developing skin cancers in Karachi.^24^ Another major risk factor could be increased production of hydochlorofluorocarbons (HCFC), leading to accelerated depletion of ozone as indicated by very high HCFC consumption that increased from 18.5 ODP metric tons in the year 1995 to 239.8 ODP metric tons in the year 2014, indicating a very high annual growth rate of 71.7%.^25^

Comparison with other regional registries show that cancer profile from registry is by and large similar to the other regional reports. However, there were some noticeable differences from Punjab Cancer Registry, particularly the high burden of cancers of lip and oral cavity in Karachi.

In summary, we report high-quality, regional cancer data representing all districts of Karachi. High frequency of preventable cancers demands serious attention by relevant authorities to improvise the existing cancer control in the country.

### Limitations of the study

While our data are representative of Karachi population, an unavoidable limitation could be a large number of cancer patients registering at other oncology/diagnostic centers of Karachi. This necessitates regular reporting of cancer data from all major stake holders to depict near-true picture of cancer burden in the city.

### Recommendations

Effective national level cancer registration system should be formulated by relevant authorities in order to delineate true picture of cancer incidence, prevalence and mortality in Pakistan. Moreover, government of Pakistan should take appropriate steps to discourage/ban use of various forms of tobacco to prevent/control tobacco-associated cancers in the country.

## CONCLUSION

Cancers of lip and oral cavity and breast cancer were the most common malignancies in males and females respectively. In children/young adults, cancers of brain and nervous system were the commonest malignancy. Alarmingly, Karachi males have highest ASR of cancers of lip and oral cavity compared to any other city of Pakistan.

### Authors’ Contribution:

**MAQ:** Founder of the cancer registry described. Conception of idea, execution and management of the whole project. Data indexation, analyses, manuscript drafting and is responsible for integrity of the study.

**SK:** Data indexation, analyses, manuscript drafting and proof reading of the manuscript**.**

**SS, MSQ:** Facilitation and Monitoring of data indexation, drafting and proof reading of manuscript.

All authors read and agreed to the final version of the manuscript.
